# Clinical Presentation and Outcome of Five Neonates With Enterovirus Central Nervous System Infection: Contrasting One Kawasaki-Like Case With Cardiac Involvement and Seizures With Four Benign Cases

**DOI:** 10.1155/crpe/5569829

**Published:** 2025-07-16

**Authors:** Alina Peternell, Christopher Schödl, Irena Odri Komazec, Matthias Baumann, Christian Lechner

**Affiliations:** ^1^Division of Pediatrics I-Pediatric Neurology, Department of Pediatric and Adolescent Medicine, Medical University of Innsbruck, Innsbruck, Austria; ^2^Division of Pediatrics III-Cardiology, Pulmonology, Allergology and Cystic Fibrosis, Department of Pediatric and Adolescent Medicine, Medical University of Innsbruck, Innsbruck, Austria

**Keywords:** central nervous system, children, enterovirus infection, myocarditis, neonates

## Abstract

**Background and Objectives:** Enteroviruses (EV) mainly cause mild infections but have been found to affect neonates more severely. The aim of this study is the description of symptoms, laboratory findings, treatment, duration of hospital stay, imaging, and outcome in five neonates presenting with EV infection of the central nervous system (CNS).

**Case Study:** All patients had signs of sepsis and/or CNS infection at first presentation and were diagnosed using cerebrospinal fluid (CSF) reverse transcriptase polymerase chain reaction (RT-PCR). One developed seizures and dilated coronary arteries and recovered after treatment with levetiracetam, intravenous immunoglobulins (IVIGs), prednisolone, and acetylsalicylic acid. This patient was also the only one to show CSF abnormalities including mononuclear pleocytosis. C-reactive protein in blood was slightly elevated in 3/5, while interleukin-6 was normal at onset and later increased (58.7–310 mg/dL) in all patients. Neutrophil-to-lymphocyte ratio was elevated (1.02–4.83) in 5/5. Antibiotics were given for 4–7 days; hospital stay lasted 7–13 days. Cerebral ultrasound was done in 2/5 and was normal in both. The patient who developed seizures underwent brain magnetic resonance imaging without pathological findings. The clinical outcome was favorable in all of our five patients.

**Conclusions:** In neonates who appear septic without an apparent focus, EV CNS infection should be considered and can be diagnosed by CSF PCR testing. Diagnosis leads to earlier discontinuation of antibiotic treatment and shorter hospital stay. Neonates with EV infection should be screened for cardiac complications and in severe cases treated with IVIG. CSF abnormalities might predict a more severe disease course and justify closer monitoring.

## 1. Introduction

Enteroviruses (EVs) mainly cause mild infections but have been found to affect neonates more severely. The most common symptoms of CNS infections in neonates are fever, irritability/lethargy, and poor feeding. Patients of all age groups may present with rash or respiratory distress, while photophobia and nuchal rigidity usually do not occur in affected neonates [1–4]. Complications such as hepatitis/hepatic necrosis with coagulopathy, severe sepsis-like syndrome, and myocarditis may lead to high morbidity and mortality [4]. Most CNS infections present as meningitis and have a usually benign course, while some patients develop encephalitis which is associated with higher mortality and long-term neurological sequelae. Signs of encephalitis include seizures, muscle hypotonia, focal neurological symptoms, altered mental status, and coma [1, 4]. Cerebrospinal fluid (CSF) reverse transcriptase polymerase chain reaction (RT-PCR) is a sensitive, specific, and fast tool to detect EV CNS infection and is, therefore, diagnostic gold standard [1, 2]. In suspected meningitis, empiric antibiotic therapy should be initiated immediately after lumbar puncture [3]. Antibiotic treatment can be discontinued once a positive PCR result is obtained and the clinical picture and laboratory findings indicate that bacterial meningitis is unlikely [1, 5]. Treatment of EV infection is mostly limited to symptomatic management. Early intravenous immunoglobulins (IVIGs) may reduce lethality rates in myocarditis and hepatic necrosis; however, there have not been any studies proofing effectiveness in CNS manifestations [2, 4]. To date, there are no licensed antiviral agents or vaccinations available [2]. Diagnosis leads to earlier cessation of antibiotic treatment and shorter hospital stay [5]. While clinical outcome is favorable in the majority of patients, etiological understanding of CNS infections is essential for improvement of diagnosis and treatment in more severe cases [1, 2, 4, 5].

## 2. Case Study

### 2.1. Patients

This retrospective case series describes five neonates with EV infection of the CNS who were treated at the University Children's Hospital of Innsbruck, Austria, between March 2019 and November 2022. All patients were diagnosed using CSF RT-PCR and met the following inclusion criteria:

• Aged 28 days or younger at the time of diagnosis
• Symptoms of CNS infection
• EV detection in CSF by RT-PCR
• Full dataset available
• Record of at least one CSF and one blood sample

### 2.2. Analyzed Parameters

We evaluated the patients' age, sex, symptoms including CNS and cardiac complications, treatment of complications, CSF and blood parameters including cell count, protein, CSF/blood glucose ratio, C-reactive protein (CRP), leukocytes, thrombocytes, interleukin-6 (IL-6), neutrophil-to-lymphocyte ratio (NLR), and bacterial cultures. Furthermore, time to diagnosis, nucleic acid detection in CSF, stool, serum and throat swab, antibiotic and antiviral treatment, duration of hospital stay, gestational age at birth, birth mode, birthweight and Apgar score, electrocardiogram (ECG), imaging (cerebral ultrasound, magnetic resonance imaging (MRI), echocardiography), and clinical outcome were assessed.

## 3. Results

### 3.1. Patient 1

Presenting symptoms of our male, 10-days-old Patient 1 included lethargy, poor feeding, and subfebrile temperature. CRP in blood was not elevated and leukocytes and thrombocytes were normal. IL-6 was normal at onset and later increased (58.7 ng/L). NLR was elevated (1.02) at onset and further increased over course of the disease (1.56). He showed CSF abnormalities including mononuclear pleocytosis (94%, 479 cells/μL) and elevated protein (1428 mg/L). CSF/blood glucose ratio was 0.51. Blood and CSF cultures yielded no bacterial growth. Nucleic acid detection in CSF led to diagnosis within 2 days. Nucleic acid of EV was additionally detected in stool, not done in serum, done but not detected in throat swab. Over the course of disease, the patient developed seizures and cardiac complications. He underwent cerebral ultrasound and brain MRI examination without pathological findings. The EEG showed individual sharp-wave suspicious steep transients frontally and centrally on the left more frequently than on the right with no seizure patterns. These EEG abnormalities were no longer detectable at follow-up 3 weeks later.

The patient had elevated troponin T (89 ng/L) and NT-pro-BNP (1474 ng/L) levels while creatine kinase and myoglobin were normal. Troponin T and NT-pro-BNP levels decreased over the course of disease and troponin T was only slightly elevated (33 ng/L) and NT-pro-BNP was normal at discharge from the hospital. Laboratory parameters were not determined at checkup.

**Figure 1:**
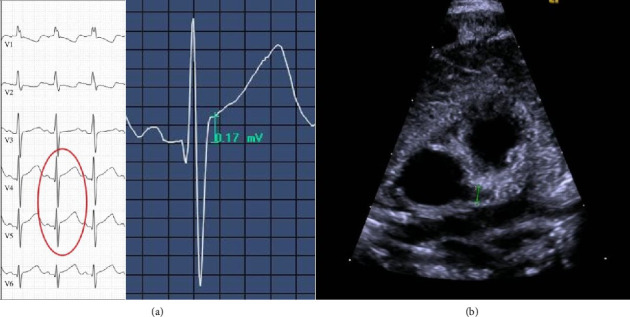
ECG of neonate with EV infection showing ST elevation in V4 and V5 (a) and echocardiography showing dilated coronary artery (b).

Echocardiography showed dilatation of the left coronary artery (in the area of the outlet, Z-score is 4.54, LAD Z-score is 2.22, and LCX could not be visualized well). The right coronary artery was minimally dilated proximally and centrally. There was no evidence of aneurysm or thrombosis (see Figure 1). Over the disease course, a second echocardiography showed the dilatation of the left coronary artery slightly (in the area of the exit, Z-score is 3.18 and LAD Z-score is 1.85). ECG showed minimal ST elevation in V4 and V5. These cardiac abnormalities were no longer detectable at checkup after 6 weeks.

He received triple antibiotic therapy consisting of ampicillin, cefuroxime, and gentamicin and additionally also acyclovir as initial treatment. Antibiotics were given for 4 days. The patient received IVIG 2 g/kg, prednisolone 2 mg/kg/d for 1 week, then 1 mg/kg/d for another week, acetylsalicylic acid 3.9 mg/kg/d for 6 weeks, levetiracetam 20 mg/kg/d for 3 weeks, and 10 mg/kg/d for another week before it was discontinued. Hospital stay lasted 13 days. The patient was born at term at a birthweight of 3332 g and had an Apgar score of 9-10-10. He was delivered by vaginal birth. The patient showed motor developmental delay at age 3 months; therefore, physiotherapy was initiated, and he was developmentally normal at 10 as well as at 18 months.

### 3.2. Patients 2–5

While Patient 1 developed seizures and cardiac complications, the remaining Patients 2–5 had a benign clinical course. They were aged 6–23 days at onset and 3/4 were boys. All patients had signs of sepsis and/or CNS infection (4/4 lethargy and poor feeding, 4/4 fever, 1/4 tachycardia, and tachypnea) at first presentation.  Table 1 shows onset symptoms and complications of our patients. They had CSF cell counts ≤ 4 cells/μL and protein < 1000 mg/L. CSF/blood glucose ratio was 0.48 and 0.55 in 2/4 and not measured in the other 2. Slightly elevated CRP in blood was found in 3/4, while leukocytes and thrombocytes were normal in all. IL-6 was elevated at onset (50.1–270 ng/L) in 4/4 and later increased (146–310 ng/L) in 3/4. NLR was elevated (1.15–4.83) in 4/4 neonates. Blood and CSF cultures yielded no bacterial growth in 4/4 patients. Nucleic acid detection in CSF led to diagnosis within 1–5 days. Nucleic acid of EV was additionally detected in stool in 2/2 evaluated patients (not done in 2 patients) and in serum in 1/1 evaluated patient (not done in 3 patients). Throat swabs were done in all patients, but EV was detected in 0/4. All patients received triple (3/4) or dual (1/4) antibiotic therapy consisting of ampicillin (4/4), cefuroxime/cefotaxime (3/4), and/or gentamicin (3/4). Antibiotics were given for 6–7 days; hospital stay lasted 7–10 days. All patients were born at term, birthweight ranged from 2965 to 3435 g, and all had an Apgar score of 9-10-10. Three neonates were vaginal births, and one was born by cesarean section. Cerebral ultrasound was done in one and was normal. Echocardiography was performed in one and showed a persistent foramen ovale with accelerated left–right shunt without hemodynamic relevance, but no sign of myocarditis. All patients developed normally at age 14, 15, 17, and 58 months, respectively.

## 4. Discussion

All patients in our cohort presented lethargic/irritable with poor feeding, 4/5 had fever, and 1/5 subfebrile temperature. This observation is supported by a large UK study including 668 patients with EV meningitis aged under 3 months in whom fever was the most common presentation (85%), followed by irritability (66%), reduced feeding (54%), and lethargy (36%) [5]. Patient 1 developed seizures and was the only one to show CSF abnormalities including mononuclear pleocytosis. Studies have found CSF pleocytosis is typical in EV CNS infection but less common in neonates [5]. Slightly elevated CRP in blood was found in 3/5, while leukocytes and thrombocytes were normal in 5/5. IL-6 was elevated at onset in 4/5 and later increased in 4/5 patients. NLR was elevated in 5/5. While similar results for CRP, leukocytes and thrombocytes have been reported [5], we could not find any studies assessing IL-6 and NLR in neonatal EV CNS infection.

Blood and CSF cultures yielded no bacterial growth in 5/5. Concurrent bacterial infection in patients with positive EV PCR result has been reported but is very rare [1]. All patients received triple (4/5) or dual (1/5) antibiotic therapy consisting of ampicillin (5/5), cefuroxime/cefotaxime (4/5), and/or gentamicin (4/5) and in one case with pleocytosis also acyclovir as initial treatment. Antibiotics were given for 4–7 days; hospital stay lasted 7–13 days. In suspected neonatal CNS infection, antibiotic treatment should be initiated directly after lumbar puncture as mortality rates in bacterial meningitis increase by the hour. Recommended agents include cefuroxime/cefotaxime and ampicillin. In some cases, aminoglycosides (such as gentamicin) or meropenem and in case of encephalitis, acyclovir should be additionally administered [3]. Antibiotic treatment may be discontinued once a positive EV PCR result is obtained and the clinical picture and laboratory findings (low CRP and leukocytes in blood, negative CSF bacterial cultures) indicate that bacterial meningitis is unlikely. It has been shown that EV PCR testing leads to shorter duration of antibiotic treatment and shorter hospital stay [1, 5].

Cerebral ultrasound was done in 2/5 and was normal in both. The patient who developed seizures underwent brain MRI without pathological findings. There are no characteristic neuroimaging abnormalities in EV CNS infection; however, brain, brainstem, and spinal cord can be affected. Generally, protocols on when to perform ultrasound and/or MRI in neonates with EV CNS infection vary greatly between countries and centers [1, 5]. In the abovementioned UK cohort, cranial ultrasound was performed in 8% (56/668) of EV meningitis cases and abnormalities were found in 5/56 (2/5 had resolving grade 1 intraventricular hemorrhage and 3/5 had small choroid plexus bleeds) while 3% (17/668) of EV meningitis cases underwent MRI examination and 8/17 were abnormal (3/8 had white matter changes and one case each of subdural hemorrhage, posterior fossa blood collection, acute infarct in the right corona radiata, severe cystic encephalomalacia, and mild ventricular dilatation) [5]. EEG was performed and showed individual sharp-wave suspicious steep transients frontally and centrally on the left more frequently than on the right with no seizure patterns. We initiated treatment with levetiracetam. Recent literature recommends levetiracetam and phenobarbital as primary agents in neonatal seizures with levetiracetam showing lesser side effects [6–9].

Echocardiography was performed in 2/5 and showed dilatation of the coronary arteries in one. Dilated coronary arteries may be a sign of EV myocarditis, which is associated with increased mortality and incidence of long-term severe cardiac sequelae such as chronic heart disease and aneurysm formation [4]. In this patient, ECG was performed showing ST elevation in V4 and V5. ST elevation is a sign of a repolarization disorder—this can not only occur in myocarditis, but also in ischemia—in the case of our patient ST elevation was most likely due to increasing demand in the hyperdynamic circulation (inflammation, fever, and tachycardia). Due to the pronounced cardiac findings and the differential diagnostic possibility of vasculitis and atypical Kawasaki syndrome, treatment with IVIG, prednisolone, and acetylsalicylic acid was initiated. Follow-up echocardiography and ECG 6 weeks later showed normalized diameters of the coronary arteries and ST elevations were no longer detectable.

The effectiveness of any of these treatments in neonatal EV infection is still debated. While administration of IVIG has been found to reduce lethality rates in cardiac complications in some studies, others have reported high mortality rates in spite of treatment with IVIG [4]. A prospective study reported that the administration of IVIG has been associated with subtle clinical improvement and faster reduction of viremia and viruria [10]. One study reported successful treatment of two out of five patients with EV myocarditis [11]. Yen et al. found that early administration of IVIG may be beneficial for survival in severe cases [12]. However, a study including 146 pediatric patients, 43 of whom were neonates, found no correlation of IVIG and clinical outcome [13]. Another recent publication reported two dramatically different cases with one twin having asymptomatic EV infection while the other twin developed severe and fatal hemorrhage–hepatitis syndrome, thus also in spite of treatment with IVIG [14].

While we have found some studies assessing treatment with IVIG, we have not found any discussing treatment with prednisolone or acetylsalicylic acid in neonatal EV infection. However, in cases with myocarditis in which a differential diagnosis of vasculitis and (atypical) Kawasaki syndrome is in question, although very rare in neonates, treatment with IVIG, prednisolone, and acetylsalicylic acid should certainly be considered [15, 16].

In line with published data, clinical outcome was favorable in all our five patients. However, our most affected patient with dilated coronary arteries, seizures, and CSF pleocytosis at the baseline showed a motor developmental delay at age 3 months. Physiotherapy was recommended, and at age 10 months, he was developmentally in the range again. Finally, mortality rates are very low and long-term sequelae are extremely rare [1, 5]. The UK study reported that less than 1% of patients with EV meningitis developed severe neurological complications, 2/668 (0.003%) died, and 4/668 (0.006%) had long-term complications [5].

Limitations of our study include a small sample size and that all patient data were collected at the same center. Furthermore, diagnostic testing differed between our patients with, for instance, not all patients receiving cerebral ultrasound, brain MRI, or echocardiography. Therefore, while our case series describes the clinical course of EV CNS infection in five neonates and compares four benign cases with one complicated by seizures and cardiac involvement, our observations cannot be generalized but rather contribute to drawing a bigger picture.

## 5. Conclusion

In neonates who appear septic without an apparent focus, CNS infection, e.g., with EV, should be considered and can be diagnosed by CSF PCR testing. Diagnosis leads to earlier discontinuation of antibiotic treatment and shorter hospital stay. Neonates with EV infection should be screened for cardiac complications and in severe cases might be treated with IVIG. CSF abnormalities might be associated with a more severe disease course and justify closer monitoring. However, CSF and blood parameters need to be investigated in a larger cohort of neonates with EV infection of the CNS.

## Figures and Tables

**Table 1:** Onset symptoms and complications of neonates with EV CNS infection in our cohort.

	Lethargy	Poor feeding	Fever	Tachypnea	Tachycardia	Seizures	Cardiac complications
Patient 1	+	+	−	−	−	+	+
Patient 2	+	+	+	−	−	−	−
Patient 3	+	+	+	+	+	−	−
Patient 4	+	+	+	−	−	−	−
Patient 5	+	+	+	−	−	−	−
	5/5	5/5	4/5	1/5	1/5	1/5	1/5

## Data Availability

Any data not published within the article will be made available in anonymized form on request from any qualified investigator.

## Nomenclature

CNS: Central nervous system
CRP: C-reactive protein
CSF: Cerebrospinal fluid
ECG: Electrocardiogram
EEG: Electroencephalogram
EV: Enterovirus
IL-6: Interleukin-6
IVIG: Intravenous immunoglobulins
LAD: Left anterior descending artery
LCX: Left circumflex artery
MRI: Magnetic resonance imaging
NLR: Neutrophil-to-lymphocyte ratio
RT-PCR: Reverse transcriptase polymerase chain reaction
